# Physiologically based pharmacokinetic model to predict drug–drug interactions with the antibody–drug conjugate enfortumab vedotin

**DOI:** 10.1007/s10928-023-09877-5

**Published:** 2023-08-26

**Authors:** Mary P. Choules, Peiying Zuo, Yukio Otsuka, Amit Garg, Mei Tang, Peter Bonate

**Affiliations:** 1 Clinical Pharmacology and Exploratory Development, Astellas Pharma Global, Inc., One Astellas Way, Northbrook, IL 60062 USA; 2 Clinical Pharmacology and Exploratory Development, Astellas Pharma Global, Inc., Tokyo, Japan; 3 Quantitative Pharmacology and Disposition, Seagen Inc., South San Francisco, CA USA

**Keywords:** Enfortumab vedotin, Urothelial carcinoma, Physiologically based pharmacokinetic model, Drug interaction

## Abstract

Enfortumab vedotin is an antibody–drug conjugate (ADC) comprised of a Nectin-4–directed antibody and monomethyl auristatin E (MMAE), which is primarily eliminated through P-glycoprotein (P-gp)–mediated excretion and cytochrome P450 3A4 (CYP3A4)–mediated metabolism. A physiologically based pharmacokinetic (PBPK) model was developed to predict effects of combined P-gp with CYP3A4 inhibitor/inducer (ketoconazole/rifampin) on MMAE exposure when coadministered with enfortumab vedotin and study enfortumab vedotin with CYP3A4 (midazolam) and P-gp (digoxin) substrate exposure. A PBPK model was built for enfortumab vedotin and unconjugated MMAE using the PBPK simulator ADC module. A similar model was developed with brentuximab vedotin, an ADC with the same valine-citrulline–MMAE linker as enfortumab vedotin, for MMAE drug–drug interaction (DDI) verification using clinical data. The DDI simulation predicted a less-than-2-fold increase in MMAE exposure with enfortumab vedotin plus ketoconazole (MMAE geometric mean ratio [GMR] for maximum concentration [C_max_], 1.15; GMR for area under the time-concentration curve from time 0 to last quantifiable concentration [AUC_last_], 1.38). Decreased MMAE exposure above 50% but below 80% was observed with enfortumab vedotin plus rifampin (MMAE GMR C_max_, 0.72; GMR AUC_last_, 0.47). No effect of enfortumab vedotin on midazolam or digoxin systemic exposure was predicted. Results suggest that combination enfortumab vedotin, P-gp, and a CYP3A4 inhibitor may result in increased MMAE exposure and patients should be monitored for potential adverse effects. Combination P-gp and a CYP3A4 inducer may result in decreased MMAE exposure. No exposure change is expected for CYP3A4 or P-gp substrates when combined with enfortumab vedotin.

**ClinicalTrials.gov identifier** Not applicable.

**Supplementary Information:**

The online version contains supplementary material available at 10.1007/s10928-023-09877-5.

## Introduction

Bladder cancer is one of the most common malignancies—among which urothelial carcinoma (UC) is the predominant type—and it is associated with poor long-term prognoses in those with advanced/metastatic disease [1–3]. Most (70%) individuals with bladder cancer are older than 65 years and present with age-associated comorbidities [2, 4]. Older individuals taking multiple medications may experience drug–drug interactions (DDIs), which can lead to reduced drug efficacy or serious health risks [5–9]. Therefore, identification of potential DDIs associated with these drugs and their metabolites is essential to support safe and effective prescribing.

Enfortumab vedotin, an antibody–drug conjugate (ADC) directed against Nectin-4, is composed of a fully human anti–Nectin-4 monoclonal antibody (mAb) conjugated to a microtubule-disrupting agent, monomethyl auristatin E (MMAE), via a protease-cleavable linker; the targeted MMAE release in Nectin-4–expressing cells leads to cell-cycle arrest and cell death [10]. Enfortumab vedotin is approved for use in multiple countries for indications including treatment of adults with locally advanced/metastatic UC who have previously received standard of care platinum-based therapy and program death receptor-1/programmed death-ligand 1 (PD-1/L1) inhibitor therapies as well as in patients previously treated with one or more lines of therapy but who are ineligible for cisplatin-containing chemotherapy [10–13]. The recommended dose of enfortumab vedotin is 1.25 mg/kg (maximum 125 mg) given as an intravenous (IV) infusion over 30 min on days 1, 8, and 15 of every 28-day cycle [10].

The nonclinical pharmacokinetic (PK) data of enfortumab vedotin from an in vivo mass balance study conducted in rats concluded that MMAE delivered by enfortumab vedotin is primarily eliminated in the feces (≤ 15% of dose via urinary excretion) with unchanged MMAE excreted in feces and urine [14]. MMAE undergoes hepatic metabolism, predominantly through cytochrome P450 3A4 (CYP3A4) and is a substrate of P-glycoprotein (P-gp) [10, 14, 15]. Clinical PK data of enfortumab vedotin (EV-101; NCT02091999) showed that mean exposure, as determined by area under the concentration-time curve (AUC) for the 3 analytes measured: ADC (antibody + conjugated drug), total antibody, and MMAE increased with ascending dose [16]. Across the tested dose range (0.5–1.25 mg/kg), maximum ADC concentrations were attained approximately 30 min to 1 h after IV administration, whereas time to reach maximum MMAE concentration was approximately 1.0 to 2.8 days after IV administration [16]. A clinical DDI study with brentuximab vedotin, an ADC featuring the same valine-citrulline–MMAE linker as enfortumab vedotin but a different target (CD30 instead of Nectin-4), showed that, while ADC exposures are unaffected by concomitant rifampin (a CYP3A inducer) or ketoconazole (a CYP3A inhibitor), MMAE exposures were lower with rifampin and higher with ketoconazole. Furthermore, brentuximab vedotin did not affect exposure to midazolam, a sensitive CYP3A substrate [17].

Physiologically based PK (PBPK) modeling has become an accepted practice for predicting DDI potential when a clinical DDI study is unavailable [18]. Results of PBPK modeling studies assist in developing guidance for clinical trial design as well as to inform drug labeling [18, 19]. Several studies have used the PBPK modeling approach to assess the DDI liability of the MMAE component of ADCs [19, 20]. Although the DDI risk for the mAb component of ADCs is low [19, 21, 22], it is important to assess the DDI potential of mAb because of its potential interaction with Fcγ receptors on the effector cells, its role in inflammatory changes in drug transport proteins, or its immune-mediated changes involving cytochrome enzymes and drug transport proteins [22–25]. Previous PBPK models for ADCs treated mAb as a small molecule and directly modeled MMAE by dosing MMAE at an equivalent dose as that released by mAb [19, 20]. The simultaneous PBPK modeling of both portions of an ADC—the large mAb and the small cytotoxic molecule—using the ADC module within the Simcyp simulator has not been previously conducted. Previous efforts around ADC modeling using Simcyp did not model the mAb as a biologic or large molecule [19, 20].

The present PBPK analysis was conducted to assess the combination of CYP3A4- and P-gp–mediated DDIs for enfortumab vedotin as both a victim and perpetrator using a model that accounted for large and small molecules. We leveraged the data from clinical DDI studies conducted in brentuximab vedotin for validating our model. The objective of this analysis was to predict the effect of a combined CYP3A4 and P-gp inhibitor (ketoconazole) or a combined CYP3A4 and P-gp inducer (rifampin) on the PK of MMAE when coadministered with enfortumab vedotin. In addition, analysis was performed to predict effects of enfortumab vedotin on the exposure of CYP3A4 substrate (midazolam) and P-gp substrate (digoxin).

## Methods

A PBPK model was developed for enfortumab vedotin using clinical data from phase 1 and 2 studies. The phase 1 dose-escalation/dose-expansion study of enfortumab vedotin (EV-101; NCT02091999) [16] was conducted in previously treated patients with Nectin-4–expressing metastatic UC and other malignant solid tumors. Single IV dose data of enfortumab vedotin 1.25 mg/kg were extracted from the study for model building and single IV dose data of enfortumab vedotin 1.0 mg/kg were used for single-dose model verification [16]. In the phase 2 single-cohort study of enfortumab vedotin (EV-201; NCT03219333), 125 patients with locally advanced/metastatic UC who were previously treated with platinum and PD-1/L1 inhibitor therapy received enfortumab vedotin 1.25 mg/kg. Multiple-dose data of enfortumab vedotin 1.25 mg/kg obtained from the study were used for multiple-dose verification [10]. Data from this study were split into a model development and model verification group.

**Fig. 1:**
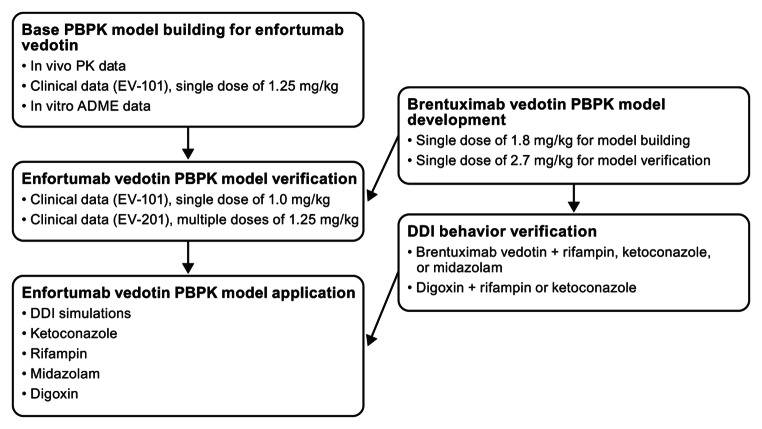
Process for PBPK model construction, verification, and application of enfortumab vedotin. *DDI*, drug–drug interaction; *IV*, intravenous; *MMAE*, monomethyl auristatin E; *PBPK*, physiologically based pharmacokinetics

Brentuximab vedotin was used in the present analysis as a baseline comparison to enfortumab vedotin because of the availability of data, including a clinical DDI trial, and similarity in the average drug–antibody ratios between these compounds. Brentuximab vedotin data, which were available from the sponsor’s clinical pharmacology submission to the US Food and Drug Administration, were digitized for model development [11]. The PK data from a single IV dose of brentuximab vedotin 1.8 mg/kg administered over 30 min were used for model building. Data from a single IV dose of brentuximab vedotin 2.7 mg/kg were used for model verification. The clinical DDI study (NCT01026415) with brentuximab vedotin and a combined P-gp and CYP3A inhibitor or inducer was used for DDI verification [17]. For verification of P-gp–mediated interactions by ketoconazole and rifampin, clinical DDI data were obtained from prior publications [12, 13]. Data were extracted from the literature using GetData Graph Digitizer version 2.26. The process for PBPK model construction, verification, and application of enfortumab vedotin is depicted in Fig. 1.

Virtual trial designs for the enfortumab vedotin and brentuximab vedotin PBPK models closely resembled the actual clinical studies [16, 17, 26]; however, for enfortumab vedotin, the default numbers of patients in the Simcyp (Certara, Sheffield, UK) simulator (10 trials with 10 patients in each trial) were used. Specifics of the virtual trial design for model construction and verification for enfortumab vedotin and brentuximab vedotin can be found in the **Supplementary Methods**.

### Model construction

**Fig. 2:**
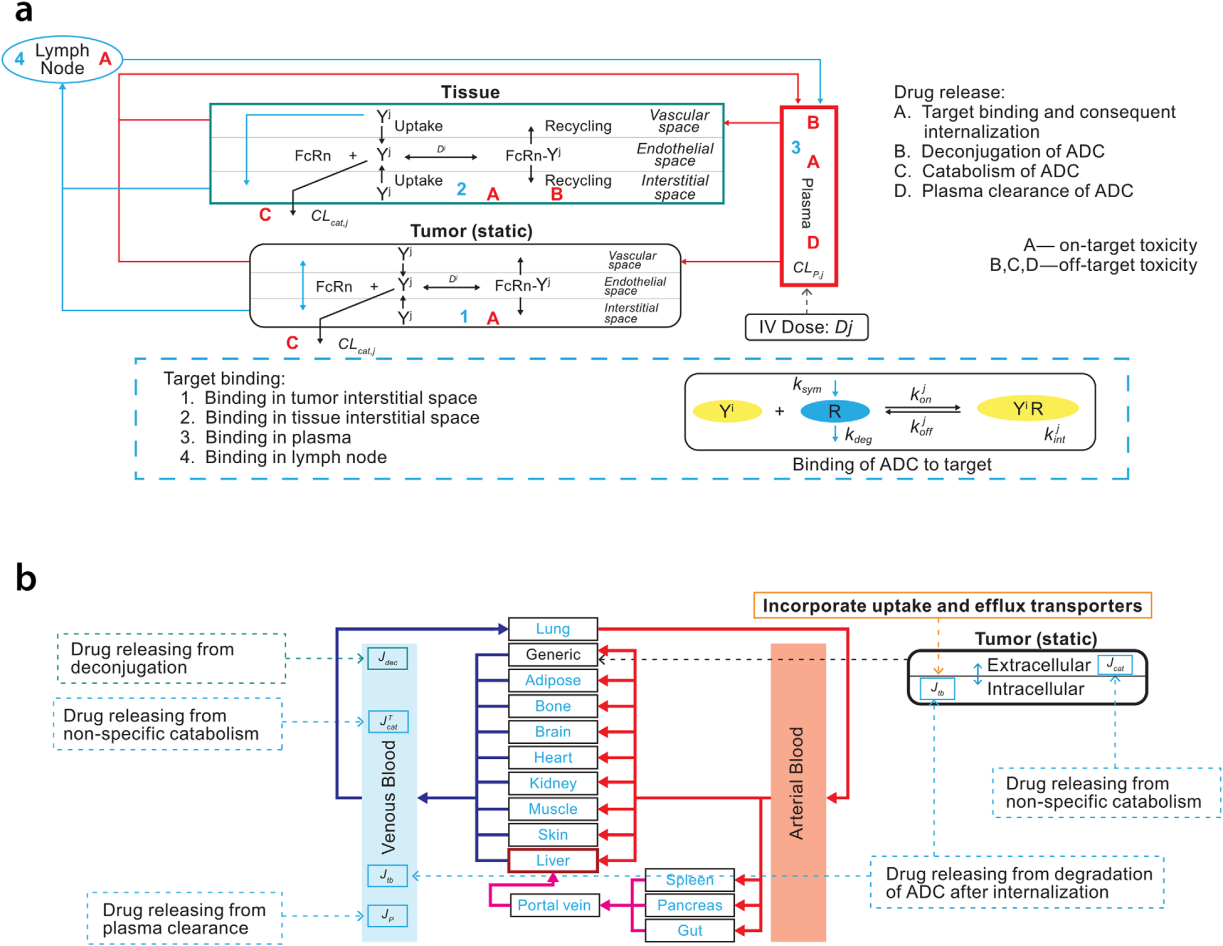
Schematic of (**a**) Simcyp simulator ADC module (**b**) linked to full PBPK model for small molecules. Reprinted with permission from Certara UK Limited. *ADC*, antibody-drug conjugate; *IV*, intravenous; *PBPK*, physiologically based pharmacokinetic model

The ADC module provided within the Simcyp simulator version 19 was used for PBPK modeling (Fig. 2). For enfortumab vedotin, the PBPK model was built using the minimal PBPK modeling approach and, for MMAE, the full PBPK approach for small molecules was used (Tables 1 and 2). The neonatal Fc receptor (FcRn) dissociation constant was optimized for enfortumab vedotin based on clinical data because an in vitro experimental value was unavailable. It was determined that the FcRn dissociation constant would be different between brentuximab vedotin and enfortumab vedotin because of the difference in mAb and that the half-life of enfortumab vedotin (~ 3.6 days for the conjugated antibody) was shorter than brentuximab vedotin (~ 4 to 6 days). A lower binding affinity (higher dissociation) to FcRn was required for enfortumab vedotin to capture the ADC elimination time curve and production of MMAE. The source of unconjugated MMAE in the model was through catabolism, additional nonspecific plasma clearance, and deconjugation. Uptake clearance and recycling rate values were unknown for enfortumab vedotin or brentuximab vedotin; due to difficulties in measuring these parameters, default values provided within the Simcyp software were used.

**Table 1:** Input parameters of the PBPK model for enfortumab vedotin using the minimal PBPK model for ADC

Parameter	Value	Source
Compound type	ADC	
Molecular weight, Da	146,664.1	IND application
Maximum DAR	8	IND application
Discrete distribution of DAR, %		
0	6.3	Automatically calculated by Simcyp (Certara, Sheffield, UK)
1	0.4	
2	28.9	
3	0.8	
4	38.9	Deposition of DAR species in enfortumab vedotin and internal study
5	1.9	
6	17.0	
7	0.6	
8	5.2	
Mean	3.735	Automatically calculated by Simcyp
FcRn binding (pH 6.0)	1:1 binding	Assumed
K_D1_ (DAR 0), µM	7.28	Optimized to clinical data
K_up_, 1/h	0.0298	Simcyp default
K_rc1_, 1/h	0.548	Simcyp default
CL_cat_ (DAR 0–8), L/h	0.0175	Simcyp default
Additional systemic CL, L/h	0.021•*j*	Assumed same as brentuximab vedotin, where *j* = DAR #
CL_lymphatic_, L/h	0	Simcyp default
K_dec, plasma_ (DAR 1), 1/h	0.001	Assumed same as brentuximab vedotin
K_dec, tissue_ (DAR 1), 1/h	0.001	Assumed same as plasma
F_rel_ (deconjugation)	1	Simcyp default and assumed
F_rel_ (catabolic)	1	Simcyp default and assumed
K_rel_ (catabolic), 1/h	1	Simcyp default and assumed

*ADC*, antibody–drug conjugate; *DAR*, drug–antibody ratio; *CL*, clearance; *CL*_*cat*_, catabolic CL; *CL*_*lymphatic*_, lymphatic CL; *F*_*rel*_, fraction released; *FcRn*, neonatal Fc receptor; *IND*, investigational new drug; *j*, DAR number; K_D1_, FcRn dissociation constant; *K*_*dec, plasma*_, plasma deconjugation rate constant; *K*_*dec, tissue*_, tissue deconjugation rate constant; *K*_*rel*_, relative rate constant; *K*_*rc1*_, recycle rate constant; *K*_*up*_, uptake rate constant; *PBPK*, physiologically based pharmacokinetic

**Table 2:** Input parameters of the PBPK model for monomethyl auristatin E using the full PBPK model for small molecules

Parameter	Value	Source
Compound type	Monoprotic base	[20]
Molecular weight, Da	717.98	[20]
Log P	2.6	[20]
pKa 1	8.08	[20]
B/P ratio	1.45	[20]
F_u_ (human serum albumin)	0.178	[20]
V_ss_, L/kg	3.03	Simcyp (Certara, Sheffield, UK) calculated, method 2, optimized with Kp scalar 0.3
CL_int_ CYP3A4, µL/min/pmol of isoform	0.00597	Simcyp retrograde calculated based on apparent CL_iv_: 2.72 L/h, f_CL, Bile_: 70%, 100% hepatic metabolic clearance CYP3A4, 0.8 CL_R_
CL_R_, L/h	0.8	Estimated based on urinary recovery and [17]
CL_int_, T ABCB1 (P-gp), µL/min/million cells	1.58	Simcyp calculated
RAF/REF	1.0	Simcyp default
CL_PD_, mL/min/million hepatocytes	0.2	Simcyp calculated based on MechPeff (Certara, Sheffield, UK)
CYP3A4 inhibition		
K_I_, µM	5	[20]
k_app_, µM	1.128	[20]
k_inact_, 1/h	6.0	[20]
ABCB1 (P-gp) competitive inhibition	—	—
Ki, µM	16.8	In vitro P-gp transporter studies

*ABCB1*, adenosine triphosphate—binding cassette transporter protein 1; *B/P*, blood–plasma ratio; *CL*_*int*_, intrinsic clearance; *CL*_*iv*_, total systemic clearance after intravenous dosing; *CL*_*PD*_, passive diffusion clearance into the hepatocyte; *CL*_*R*_, renal clearance; *CYP3A4*, cytochrome P450 3A4; *f*_*CL, Bile*_, fraction (%) clearance through the bile; *F*_*u*_, fraction of unbound parent or metabolite in plasma; *K*_*app*_, concentration that produces half maximal rate of inactivation; Ki, reversible inhibition constant; *k*_*inact*_, maximal inactivation rate constant; *Log P*, logarithm of octanol–water partition coefficient; *K*_*I*_, inhibitor concentration at 50% of k_inact_; *PBPK*, physiologically based pharmacokinetic; *P-gp*, P-glycoprotein; *pKa*, logarithmic acid dissociation constant; *RAF/REF*, relative activity factor/relative expression factor; *V*_*ss*_, volume of distribution at steady state

The MMAE compound model was constructed using values obtained from a previous study of PBPK model building of brentuximab vedotin with some modifications [20], because the previous model was built using an older version of Simcyp that did not contain the ADC module. Use of the volume of distribution at steady state and CYP3A4 intrinsic clearance reported in the reference study led to an underestimation of MMAE exposure. Thus, these parameters were optimized for the ADC module and the enfortumab vedotin PBPK model. For the brentuximab vedotin PBPK model, ADC plasma clearance was optimized using clinically observed data from phase 1 studies with brentuximab vedotin administered at 1.8 mg/kg. The same MMAE compound file was utilized for enfortumab vedotin and brentuximab vedotin (Table S1).

### Model verification

For enfortumab vedotin, the PBPK model was verified by analyzing the ADC and MMAE concentration time profiles with single-dose (from the first dose within a cycle) enfortumab vedotin 1.0 mg/kg and multiple doses of enfortumab vedotin 1.25 mg/kg from phase 1 and 2 trials, respectively [10, 16]. For brentuximab vedotin, the PBPK model was verified by the ADC and MMAE concentration time profiles with the dose of brentuximab vedotin 2.7 mg/kg from the clinical pharmacology data submitted to the US Food and Drug Administration [26].

Model verification of drug interactions with brentuximab vedotin was conducted with a combined P-gp and CYP3A4 inhibitor or inducer and CYP3A4 substrate. The simulated plasma concentration profiles, ratio of AUC from time 0 extrapolated to infinity (AUC_inf_), and ratio of maximum serum concentration (C_max_) of ADC and MMAE from brentuximab vedotin 1.2 mg/kg with ketoconazole 400 mg oral daily or brentuximab vedotin 1.8 mg/kg with rifampin 600 mg oral daily or a single IV dose of midazolam 1 mg were compared with clinical PK data from a previously published study [17]. Rifampin simulations were performed with the fold-increase of the P-gp transporter relative activity factor value applied to MMAE, as previously described.

For the cancer population, the distributions of simulated values for age, body weight, plasma albumin value, and hematocrit level were compared with those observed from all patients in the phase 1 study [16] for verification of the Simcyp simulator–provided cancer population modification.

### Model for drug–drug interaction simulations

The verified PBPK model was used to evaluate the interaction of enfortumab vedotin with ketoconazole, rifampin, midazolam, and digoxin. For the DDI simulation studies, the clinical brentuximab vedotin DDI data were leveraged to verify the applicability of the brentuximab vedotin PBPK model in predicting DDIs observed clinically. This information was then used for enfortumab vedotin to predict the exposure of MMAE in several DDI simulations.

To analyze effects of the combined P-gp and CYP3A4 inhibitor and inducer on enfortumab vedotin and brentuximab vedotin, the Simcyp simulator–provided PBPK models for ketoconazole and rifampin were slightly modified. An inhibitory constant (Ki) for the P-gp transport for intestine and liver compartments was analyzed and incorporated for both models; for ketoconazole, a P-gp inhibition K_I_ value of 0.67 µM was added to the ketoconazole PBPK compound model file; for rifampin, a K_I_ value of 4.3 µM was added. No other parameters were altered.

To analyze the inhibitory effects of enfortumab vedotin and brentuximab vedotin on CYP3A4 and P-gp substrates, the Simcyp simulator PBPK models for midazolam and digoxin were used for enfortumab vedotin and brentuximab vedotin DDI simulations. The model for midazolam was used without modification. For simulations with rifampin, the relative activity factor for P-gp transport within the digoxin compound file was increased to represent transporter induction. Rifampin simulations were performed twice: once with the increased relative activity value contained within the digoxin compound file, then again with the baseline relative activity value and rifampin dose set to 0. Of note, the state of the simulator at the time of analysis did not support transporter induction for DDI simulation; therefore, the effect of P-gp induction by rifampin was manually implemented.

The difference between enfortumab vedotin and brentuximab vedotin populations was hypothetically linked to the included cancer population (solid tumor vs. blood cancer) within the clinical studies [16, 26]. The Simcyp cancer population model was used without modification for all brentuximab vedotin simulations. For simulations of enfortumab vedotin, the population was slightly modified and the influence of the tissue–volume scaling factor for plasma on the ADC plasma concentrations was investigated. For digoxin simulations, the Simcyp simulator–containing PBPK model for the healthy volunteer population was used without modifications.

### Application of the drug–drug interaction simulation and sensitivity analysis

The verified enfortumab vedotin PBPK model was employed to simulate interactions between enfortumab vedotin and rifampin, ketoconazole, midazolam, and digoxin using the virtual trial design (Table S2). Sensitivity analysis was conducted to evaluate the uncertainty of P-gp (biliary) versus CYP3A4 contribution on elimination of MMAE. The drug interactions between enfortumab vedotin and ketoconazole or rifampin were used to analyze the effect of the elimination pathway on the GMRs of MMAE C_max_ and AUC from time 0 to last quantifiable concentration (AUC_last_; information regarding times for AUC_last_ appears in Table S2). Simulations were run for a sufficient time duration for the AUC_last_ ratio to be equivalent to the AUC_inf_ ratio.

### Statistical analysis

Summary statistics of simulated C_max_, AUC_last_, AUC_inf_, AUC from time 0 to day 7 (AUC_d0–7_), AUC from time 0 to day 14 (AUC_d0–14_), C_max_ ratio, and AUC_inf/last_ ratio were calculated by noncompartmental analysis. For DDI simulations involving rifampin, midazolam, and digoxin, geometric mean ratios (GMRs) were calculated for C_max_ and AUC_last_ (reported instead of AUC_inf_ due to limitations in calculating AUC_inf_ for some simulations). The GMR was calculated from the 2 simulations using R-Studio (R-Studio, Boston, MA).

## Results

### Enfortumab vedotin

**Fig. 3:**
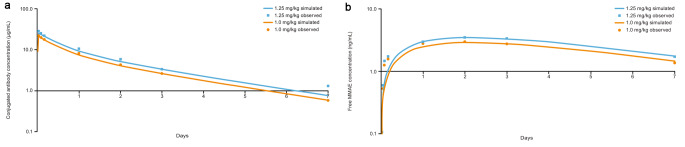
Simulated versus observed semilog plasma concentration curves of (**a**) conjugated enfortumab vedotin and (**b**) MMAE following IV administration of enfortumab vedotin 1.0 mg/kg and 1.25 mg/kg. *IV*, intravenous; *MMAE*, monomethyl auristatin E

**Fig. 4:**
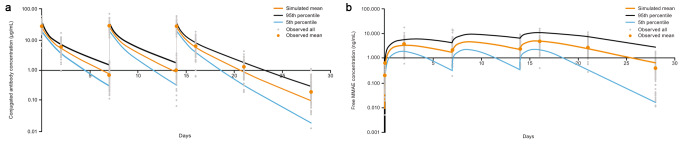
Simulated versus observed semilog plasma concentration curves of (**a**) conjugated enfortumab vedotin and (**b**) MMAE following multiple IV administration of 1.25 mg/kg. *IV*, intravenous; *MMAE*, monomethyl auristatin E

The simulation reproduced the observed plasma concentration-time profile and PK parameters for enfortumab vedotin (ADC) and MMAE from the phase 1 clinical study for enfortumab vedotin (Table 3) [16]. Simulated concentration-time profiles aligned with observed concentration-time profiles for a single dose of enfortumab vedotin 1.0 and 1.25 mg/kg at all time points for enfortumab vedotin and from days 2 to 7 for MMAE (Fig. 3). Similarly, for multiple doses of enfortumab vedotin 1.0 and 1.25 mg/kg, the simulation reproduced the observed concentration-time profile (Fig. 4) and PK parameters for enfortumab vedotin (ADC) and MMAE (Table 4). Thus, for enfortumab vedotin, the PBPK model reasonably characterized the observed PK.

**Table 3:** Comparison of observed and predicted enfortumab vedotin and MMAE PK parameters^a^

Enfortumab vedotin PK parameter	Observed			Simulated		
	AUC _d0–7_, µg•day/mL	C_max_, µg/mL	t_1/2_, day	AUC _d0–7_, µg•day/mL	C_max_, µg/mL	t_1/2_, day
1.25 mg/kg						
n	32	38	33	100	100	100
Mean (SD)	34.5 (7.78)	29.1 (8.23)	1.67 (0.314)	32.6 (5.12)	25.3 (4.63)	1.96 (0.280)
GM	33.7	28.2	1.64	32.2	24.9	1.94
CV%	22.5	28.2	18.8	15.7	18.3	14.3
1.0 mg/kg						
n	25	27	28	100	100	100
Mean (SD)	28.5 (5.99)	22.7 (4.54)	1.70 (0.264)	26.0 (4.15)	20.6 (3.91)	1.94 (0.283)
GM	27.9	22.3	1.68	25.7	20.2	1.93
CV%	21.0	20.0	15.6	16.0	19.0	14.5
MMAE PK parameter	Observed			Simulated		
	AUC _d0–7_, ng•day/mL	C_max_, ng/mL	t_1/2_, day	AUC _d0–7_, ng•day/mL	C_max_, ng/mL	t_1/2_, day
1.25 mg/kg						
n	16	37	2	100	100	100
Mean (SD)	19.5 (12.8)	3.59 (2.11)	4.11 (—)	18.5 (8.60)	3.52 (1.41)	3.37 (1.76)
GM	16.0	3.03	—	16.8	3.28	3.05
CV%	65.9	58.7	—	46.4	40.1	52.3
1.0 mg/kg						
n	15	29	4	100	100	100
Mean (SD)	15.1 (7.95)	2.91 (1.49)	2.93 (0.198)	15.5 (7.44)	2.93 (1.22)	3.34 (1.54)
GM	13.3	2.58	2.93	14.0	2.72	3.06
CV%	52.5	51.1	6.8	48.1	41.6	46.1

*AUC*_*d0*–*7*_, area under the concentration-time curve from time 0 to 7 day postdose; *C*_*max*_, maximum concentration; *CV*, coefficient of variation; *GM*, geometric mean; *MMAE*, monomethyl auristatin E; *PK*, pharmacokinetic; *SD*, standard deviation; t_1/2_, terminal half-life

^a^Data for single dose

**Table 4:** Comparison of observed and predicted pharmacokinetic parameters following multiple intravenous doses of enfortumab vedotin 1.25 mg/kg

Parameter	Observed				Simulated			
	Day 1		Day 15		Day 1		Day 15	
	AUC_7d_, µg•day /mL	C_max_, µg/mL	AUC_14d_, µg•day /mL	C_max_, µg/mL	AUC_7d_, µg•day /mL	C_max_, µg/mL	AUC_14d_, µg•day /mL	C_max_, µg/mL
Enfortumab vedotin								
n	134	143	93	112	150	150	150	150
Mean (SD)	38.1 (11.4)	27.4 (7.7)	42.4 (16.0)	26.5 (7.6)	32.8 (5.56)	25.4 (4.69)	37.8 (8.08)	26.0 (4.68)
GM	36.4	26.2	39.5	25.5	32.4	25.0	36.9	25.6
CV%	30.0	28.3	37.7	28.5	16.9	18.5	21.4	18.0
	Observed				Simulated			
	Day 1		Day 15		Day 1		Day 15	
	AUC_7d_, ng•day/mL	C_max_, ng/mL	AUC_14d_, ng•day/mL	C_max_, ng/mL	AUC_7d_, ng•day/mL	C_max_, ng/mL	AUC_14d_, ng•day/mL	C_max_, ng/mL
MMAE								
n	130	139	98	110	150	150	150	150
Mean (SD)	17.6 (11.9)	3.7 (2.4)	34.0 (23.8)	4.7 (2.9)	18.1 (8.71)	3.42 (1.42)	36.6 (29.1)	5.00 (2.8)
GM	14.6	3.0	28.0	4.0	16.4	3.19	29.2	4.43
CV%	67.8	65.5	70.1	61.2	48.2	41.4	79.4	56.1

Observed data are from the phase 2 trial

*AUC*_*7d*_, area under the concentration-time curve from time 0 to 7 days postdose; *AUC*_*14d*_, area under the concentration-time curve from time 0 to 14 days postdose; *C*_*max*_, maximum concentration; *CV*, coefficient of variation; *GM*, geometric mean; *MMAE*, monomethyl auristatin E; *SD*, standard deviation

### Brentuximab vedotin

For brentuximab vedotin 1.8 and 2.7 mg/kg, the simulation aligned with the observed concentration-time profile for a single dose (Figure S1) and PK parameters for brentuximab vedotin (ADC) and MMAE (Table S3). The DDI simulation of brentuximab vedotin with ketoconazole adequately reproduced the observed fold-change in AUC and C_max_ (Table 5). The simulated DDI fold-change in AUC_last_ when brentuximab vedotin was dosed with rifampin or midazolam was slightly overestimated; however, the difference was deemed acceptable. No additional optimization was deemed necessary for the MMAE elimination pathway or CYP3A4 inhibition constant, and the MMAE CYP3A4 and P-gp pathway was verified.

**Table 5:** Observed and predicted pharmacokinetic effects of monomethyl auristatin E for enfortumab vedotin or brentuximab vedotin in the presence of ketoconazole, rifampin, midazolam, or digoxin

Parameter		Observed [17]		Predicted		Predicted/Observed ratio	
		AUC_inf_ ratio	C_max_ ratio	AUC_inf_ ratio	C_max_ ratio	AUC_inf_	C_max_
Ketoconazole (combined P-gp and strong CYP3A inhibitor)							
Brentuximab vedotin 1.8 mg/kg	GM	1.34	1.25	1.37	1.15	1.02	0.92
	90% CI	0.98–1.84	0.90–1.72	1.35–1.39	1.14–1.16	—	—
Enfortumab vedotin 1.25 mg/kg	GM	—	—	1.38	1.15	—	—
	90% CI	—	—	1.35–1.41	1.14–1.16	—	—
Rifampin (combined P-gp and strong CYP3A inducer)							
Brentuximab vedotin 1.8 mg/kg	GM	0.54	0.56	0.47	0.70	0.89	1.25
	90% CI	0.43–0.68	0.42–0.76	0.46–0.49	0.69–0.70	—	—
Enfortumab vedotin 1.25 mg/kg	GM	—	—	0.47	0.72	—	—
	90% CI	—	—	0.46–0.49	0.71–0.73	—	—
Midazolam (CYP3A4 substrate)							
Brentuximab vedotin 1.8 mg/kg	GM	0.94	1.15	1.20	1.00	1.28	0.87
	90% CI	0.81–1.10	0.76–1.74	1.18–1.21	1.00–1.00	—	—
Enfortumab vedotin 1.25 mg/kg	GM	—	—	1.14	1.00	—	—
	90% CI	—	—	1.13–1.16	1.00–1.00	—	—
Digoxin (P-gp substrate)							
Brentuximab vedotin 1.8 mg/kg	GM	—	—	1.00	1.00	—	—
	90% CI	—	—	1.00–1.00	1.00–1.00	—	—
Enfortumab vedotin 1.25 mg/kg	GM	—	—	1.00	1.00	—	—
	90% CI	—	—	1.00–1.00	1.00–1.00	—	—

*AUC*_*inf*_, area under the time-concentration curve from time to infinity; *AUC*_*last*_, area under the time-concentration curve from time to the last measurable concentration; *CI*, confidence interval; *C*_*max*_, maximum plasma concentration; *CYP3A*, cytochrome P450 3 A; *GM*, geometric mean; *P-gp*, P-glycoprotein

### Cancer population

The base cancer population within the Simcyp simulator was modified for enfortumab vedotin simulations by changing the tissue–volume scaling factor for plasma from 1.2 to 1.0 due to underestimation of C_max_ in conjugated enfortumab vedotin plasma concentrations. The simulated population closely resembled the observed patient population, and the population demographics were generally similar (Table S4). The model was determined to be biologically plausible for combined P-gp and CYP3A4 drug-interaction simulations because it successfully re-created clinically observed plasma–concentration data (Table 6) and because similar results were observed in clinical DDI studies performed with brentuximab vedotin, which has the same cytotoxic agent and drug linker as enfortumab vedotin.

**Table 6:** Summary of ADC predicted vs. observed pharmacokinetic parameters

ADC	Reference	Dose, mg/kg	C_max_, µg/mL			AUC, µg•day/mL		
			Predicted	Observed	P/O	Predicted^a^	Observed^b^	P/O
Conjugated antibody								
Brentuximab vedotin	[11, 27]	1.8	30.9	32.0	0.966	79.7	79.4	1.00
Brentuximab vedotin	[11, 27]	2.7	46.4	45.0	1.031	120	126	0.952
Enfortumab vedotin	[16]	1.25	24.9	28.2	0.883	32.2	33.7	0.955
Enfortumab vedotin	[16]	1.00	20.2	22.3	0.906	25.7	27.9	0.921
		Dose, mg/kg	C_max_, ng/mL			AUC, ng•day/mL		
			Predicted	Observed	P/O	Predicted	Observed	P/O
MMAE								
Brentuximab vedotin	[11, 27]	1.8	4.28	4.67	0.916	32.0	37.0	0.865
Brentuximab vedotin	[11, 27]	2.7	6.44	7.00	0.920	48.6	53.2	0.914
Enfortumab vedotin	[16]	1.25	3.28	3.03	1.08	16.8	16.0	1.050
Enfortumab vedotin	[16]	1.00	2.72	2.58	1.05	14	15.1	0.927

Data expressed as geometric mean; citations for observed data

*ADC*, antibody–drug conjugate; *AUC*, area under the concentration-time curve; *C*_*max*_, maximum concentration; *MMAE*, monomethyl auristatin E; *P/O*: predicted/observed

^a^Expressed as AUC from 0 to infinity

^b^Expressed as AUC from 0 to last measured point

### Prediction of drug–drug interaction with enfortumab vedotin and sensitivity analysis

The GMRs for C_max_ and AUC_last_ of MMAE from enfortumab vedotin in the presence of ketoconazole were 1.15 (15% increase) and 1.38 (38% increase) respectively. (Table 5). The GMRs for C_max_ and AUC_last_ of MMAE in the presence of rifampin were 0.72 (28% decrease) and 0.47 (53% decrease), respectively, indicating moderate impact on MMAE plasma exposure. The GMRs of midazolam in the presence of enfortumab vedotin were 1.00 for C_max_ and 1.14 for AUC_last_ and the GMRs of digoxin in the presence of enfortumab vedotin were 1.00 for both C_max_ and AUC_last_, predicting no effect on digoxin exposure in the presence of enfortumab vedotin, thus indicating that enfortumab vedotin has minimal impact on CYP3A4 or P-gp substrates. These findings were similar to the fold-difference clinically observed and simulated for brentuximab vedotin.

Considering the potential range of biliary excretion, sensitivity analyses indicated that a 10% difference in the assumed fraction clearance through the bile had limited impact on the magnitude of DDI simulation results when enfortumab vedotin 1.25 mg/kg was coadministered with ketoconazole 400 mg or rifampin 600 mg (Table S5).

## Discussion

Physiologically based PK is a mechanistic modeling framework that has become an important tool for PK and DDI prediction for drug development [18, 28]. In this study, a PBPK model was built to predict the MMAE-based DDI for enfortumab vedotin and effect of enfortumab vedotin on CYP3A4 and P-gp substrates; the feasibility of using the Simcyp ADC module for PBPK modeling was also demonstrated. Based on the results of this study, patients receiving enfortumab vedotin with combination P-gp and CYP3A4 inhibitors may experience increases in MMAE exposure. The predicted increase in AUC with concomitant administration of enfortumab vedotin and ketoconazole was less than 2-fold. At the recommended clinical dose of enfortumab vedotin 1.25 mg/kg, there was minimal interpatient variability in enfortumab vedotin exposure. However, the interpatient variability in MMAE exposure was greater than 2-fold; therefore, a less-than-2-fold increase in plasma exposure can be considered within interpatient variability. Regardless, patients should be monitored for adverse reactions. Patients may also receive concurrent treatment with enfortumab vedotin and combined P-gp and strong CYP3A4 inducers may experience a decrease in exposure to MMAE. The predicted decrease in unconjugated MMAE AUC with concomitant administration of rifampin was greater than 50% but less than 80%, suggesting a moderate impact of rifampin on the plasma exposure of MMAE.

With regards to enfortumab vedotin being a perpetrator of drug interactions of CYP3A or P-gp substrates, the predicted increase in midazolam AUC and no change in digoxin AUC indicated that enfortumab vedotin has minimal impact on these substrates. Hence, no dose adjustments would be required for CYP3A or P-gp substrates when concomitantly used with enfortumab vedotin.

In the present study, PBPK modeling was based on the advanced Simcyp “ADC” module feature, which simultaneously simulates the large mAb and the cytotoxic small molecule. Unlike previous studies, the PBPK model used in this study considered the effect of P-gp on the resulting DDIs, whereas previous studies did not [17, 19, 20]. The PBPK model using the Simcyp “ADC” module feature allows researchers to study specific populations of virtual patients to assess their likely response to a specific ADC. In addition, this PBPK approach facilitates mechanism-driven modeling, characterization, and simulation studies of ADCs and DDIs. The present work was used in lieu of a dedicated clinical study. Similar results to the clinical DDI studies for brentuximab vedotin were demonstrated based on predicted/observed ratios reported in Table 5. In addition, the model successfully re-created clinically observed plasma concentration data (Table 6). Extrapolation of the model for brentuximab vedotin to enfortumab vedotin was rational because both ADCs contain MMAE and the same linker; thus, the model was highly biologically plausible for the combined P-gp and CYP3A DDI simulations.

In terms of model limitations, because of limited data, assumptions were made when creating the conjugated mAb portion of the enfortumab vedotin model. At the time this study was conducted, antibody conjugated MMAE measurements were unavailable. Moreover, MMAE concentration-time profiles displayed large variability that could not be fully captured by the model. Association with conjugation and changes in FcRn binding has been reported in the literature and there are instances where conjugation increases or decreases FcRn binding [29, 30]. Binding of brentuximab vedotin to FcRn was obtained from the literature and experimental values [31]. Because an in vitro experimental value for the binding affinity of enfortumab vedotin to the FcRn was unknown at the time of modeling, values were optimized based on observed clinical data; however, this limitation did not affect the ability of the model to evaluate the drug interaction potential of enfortumab vedotin. Considering the similarity in the linker and MMAE, it was assumed that the deconjugation rate and additional plasma clearance were the same as brentuximab vedotin. A worst-case scenario, with the assumption that the excretion of MMAE through transport was limited only by the clearance of P-gp, was used for the passive diffusion of MMAE across the hepatocytes prior to P-gp export. Due to lack of available data, target-mediated drug disposition (TMDD) could not be included in the present model and no interpretations of the role of TMDD on enfortumab vedotin distribution and elimination could be made. Although it would be expected that MMAE exposure in tumors should be greater than in healthy tissue given the greater expression of Nectin-4 in tumors versus healthy tissue [32–34], predictions of drug interactions as a result of target exposure in tissues expressing Nectin-4 (i.e., MMAE concentration in healthy tissue vs. tumors) were not studied.

## Conclusion

Based on the results of the PBPK simulation for enfortumab vedotin, no dose adjustment is required for concomitant administration of enfortumab vedotin when used in combination with a P-gp and inhibitors or inducers of CYP3A4. However, patients receiving enfortumab vedotin with combined P-gp and CYP3A4 inhibitors may experience increases in MMAE exposure and should be monitored for signs of adverse events. No change in exposure is expected with CYP3A4 or P-gp substrates for patients concomitantly receiving these substrates with enfortumab vedotin. This work underpins the applicability of PBPK modeling to predict ADC drug interactions and demonstrates that MMAE associated with enfortumab vedotin has limited potential for causing clinically relevant DDIs.

## Electronic supplementary material

Below is the link to the electronic supplementary material.

Supplementary Material 1

## Data Availability

Researchers may request access to anonymized participant level data, trial level data and protocols from Astellas sponsored clinical trials at www.clinicalstudydatarequest.com. For the Astellas criteria on data sharing see: https://clinicalstudydatarequest.com/Study-Sponsors/Study-Sponsors-Astellas.aspx. Researchers may request access to the Simcyp files used to support this article by contacting the corresponding author.
