# Supplementary material for: Physiologically based pharmacokinetic model to predict drug–drug interactions with the antibody–drug conjugate enfortumab vedotin
Source: J Pharmacokinet Pharmacodyn. 2023 Aug 26;51(5):417–28. doi: 10.1007/s10928-023-09877-5 (PMC11576838; doi:10.1007/s10928-023-09877-5)
Supplement: Supplementary file 1 — Supplementary Material 1 [file 10928_2023_9877_MOESM1_ESM.docx]

**SUPPLEMENTARY INFORMATION**

**Physiologically based pharmacokinetic model to predict drug–drug interactions with the antibody–drug conjugate enfortumab vedotin**

Mary P. Choules,^1,^* Peiying Zuo,^1^ Yukio Otsuka,^2^ Amit Garg,^3^ Mei Tang,^1^ Peter Bonate^1^

^1^Clinical Pharmacology and Exploratory Development, Astellas Pharma Global Development, Inc., Northbrook, IL; ^2^Clinical Pharmacology and Exploratory Development, Astellas Pharma Global Development, Inc., Tokyo, Japan; ^3^Quantitative Pharmacology and Disposition, Seagen Inc., South San Francisco, CA

**Corresponding Author:**

Phone: 847-224-3252

Email: mary.choules@astellas.com

**Methods**

**Virtual trial design for physiologically based pharmacokinetic model construction and verification for enfortumab vedotin**

The virtual trial design for the physiologically based pharmacokinetic (PBPK) model simulations used individuals with cancer aged 24 to 83 years (women, 26%) for enfortumab vedotin 1.25 mg/kg (single intravenous [IV] dose over 30 minutes); for trials with 1.0-mg/kg dosing, the patients in the cancer population were aged 48 to 81 years (women, 37%). In total, 10 trials (default) with 10 participants (default) each were tested. A total of 800 samples was collected over a study duration of 168 hours. To verify the enfortumab vedotin PBPK model, a virtual trial design with patients in the cancer population aged 40 to 90 years (women, 29%) received multiple-dose IV administration over 30 minutes of enfortumab vedotin 1.25 mg/kg. In total, 10 trials (default) with 15 participants each were tested. A total of 3000 samples was used for a study duration of 672 hours. The virtual trial designs for the enfortumab vedotin PBPK model resembled the actual clinical studies as closely as possible.

**Virtual trial design for model construction and verification for brentuximab vedotin**

The virtual trial design for the PBPK model simulations used individuals with cancer aged 22 to 70 years (assumed; women, 40% [assumed]) for brentuximab vedotin 1.8 or 2.7 mg/kg (single IV dose over 30 minutes). In total, 10 trials (default) with 12 participants each were tested. A total of 2000 samples was used for a study duration of 504 hours. The virtual trial designs for the brentuximab vedotin PBPK model resembled the actual clinical studies as closely as possible.

**Table S1** Input parameters of PBPK model for brentuximab vedotin using minimal PBPK model for ADC

| Parameter | Value | Source |
| --- | --- | --- |
| Compound type | ADC |  |
| Molecular weight, Da | 148,081 | [1] |
| Maximum DAR | 8 | [2] |
| Discrete distribution of DAR, % |  |  |
| 0 | 5.8 | Automatically calculated by Simcyp (Certara, Sheffield, UK) |
| 1 | 0 | [3] |
| 2 | 27.5 | [3] |
| 3 | 0 | [3] |
| 4 | 35.5 | [3] |
| 5 | 0 | [3] |
| 6 | 22.0 | [3] |
| 7 | 0 | [3] |
| 8 | 9.2 | [3] |
| Mean | 4.026 | Automatically calculated by Simcyp |
| FcRn binding (pH 6.0) | 2:1 binding | [3] |
| K_D1_ (DAR 0), μM | 2.47 × 10^−4^ | [3] |
| K_D2_ (DAR 0), μM | 3.23 × 10^−5^ | [3] |
| K_up_, 1/h | 0.0298 | Simcyp default |
| K_rc1_, 1/h | 0.548 | Simcyp default |
| K_rc2_, 1/h | 1.125 | Simcyp default |
| CL_cat_ (DAR 0–8), L/h | 0.0175 | Simcyp default |
| Additional systemic CL, L/h | 0.021•*j* | Best fit, where j=DAR # |
| CL_lymphatic_, L/h | 0 | Simcyp default |
| K_dec, plasma_ (DAR 1), 1/h | 0.001 | [4] |
| K_dec, tissue_ (DAR 1), 1/h | 0.001 | Assumed same as plasma |
| F_rel_ (deconjugation) | 1 | Simcyp default and assumed |
| F_rel_ (catabolic) | 1 | Simcyp default and assumed |
| K_rel_ (catabolic), 1/h | 1 | Simcyp default and assumed |

*ADC*, antibody–drug conjugate; *AR*, drug–antibody ratio; *CL*, clearance; *CL_cat_*, catabolic CL; *CL_lymphatic_*, lymphatic CL; *DAR*, drug-antibody ratio; *FcRn*, neonatal Fc receptor; *F_rel_*, fraction released; *j*, DAR number; *K_D_*, dissociation constant; *K_dec, plasma_*, plasma deconjugation rate constant; *K_dec, tissue_*, tissue deconjugation rate constant; *K_rc,_* recycle rate constant; *K_rel_*, relative rate constant; K_up_, uptake rate constant; *PBPK*, physiologically based pharmacokinetic

**Table S2** Virtual trial design for drug–drug interaction model application for enfortumab vedotin and ketoconazole, rifampin, midazolam, or digoxin

| Parameter | Number/Selected option | | | |
| --- | --- | --- | --- | --- |
|  | Ketoconazole | Rifampin | Midazolam | Digoxin |
| Virtual trial population | Cancer population model within Simcyp (Certara, Sheffield, UK) | | | |
| Trials, n | 10 | | | |
| Patients in each trial, n | 10 (default) | | | |
| Women, % | 30 | | | |
| Age, y | 40–90 | | | |
| Food condition | Fasted | | | |
| Object drug | Single IV dose of enfortumab vedotin 1.25 m/kg over 30 min | | | |
| Candidate inducer or inhibitor of P-gp and/or CYP3A | 400 mg daily | 600 mg daily | 1-mg IV bolus over 2 min | 1-mg IV over 30 min |
| Dosing scheme | Enfortumab vedotin day 4 with ketoconazole daily | Enfortumab vedotin day 8 with rifampin daily | Enfortumab vedotin day 4 with midazolam on days 1 and 6 | Enfortumab vedotin day 1 with digoxin on day 3 |
| Study duration, h | 984 | 936 | 600 | 336 |
| Sample, n | 4000 | 3000 | 2500 | 1400 |

*CYP3A*, cytochrome P40 3A; *IV*, intravenous; *P-gp*, P-glycoprotein

**Table S3** Comparison of observed and predicted brentuximab vedotin and MMAE pharmacokinetic parameters of brentuximab vedotin

| Brentuximab vedotin, variable | Observed | | | Simulated | | |
| --- | --- | --- | --- | --- | --- | --- |
|  | AUC_inf_, μg•day/mL | C_max_, μg/mL | t_1/2_, day | AUC _inf_, μg•day/mL | C_max_, μg/mL | t_1/2_, day |
| 1.8 mg/kg | | | | | | |
| n | 12 | 12 | 12 | 120 | 120 | 120 |
| Mean (SD) | — | — | — | 82.2 (20.4) | 31.5 (6.09) | 5.71 (1.33) |
| GM | 79.4 | 32.0 | 4.43 | 79.7 | 30.9 | 5.56 |
| CV% | 30 | 29 | 38 | 24.8 | 19.3 | 23.2 |
| 2.7 mg/kg | | | | | | |
| n | 12 | 12 | 12 | 120 | 120 | 120 |
| Mean (SD) | — | — | — | 123 (30.6) | 47.2 (9.14) | 5.71 (1.33) |
| GM | 126 | 45.0 | 5.98 | 120 | 46.4 | 5.56 |
| CV% | 19 | 16 | 30 | 24.8 | 19.3 | 23.2 |
| MMAE, variable | Observed | | | Simulated | | |
|  | AUC_inf_, ng•day/mL | C_max_, ng/mL | t_1/2_, day^a^ | AUC _inf_, ng•day/mL | C_max_, ng/mL | t_1/2_, day^a^ |
| 1.8 mg/kg | | | | | | |
| n | 12 | 12 | 12 | 120 | 120 | 120 |
| Mean (SD) | — | — | — | 38.5 (27.3) | 4.62 (1.99) | 3.57 (0.772) |
| GM | 37.0 | 4.97 | 3.60 | 32.0 | 4.28 | 3.50 |
| CV% | 47 | 43 | 25 | 71.0 | 43.1 | 21.6 |
| 2.7 mg/kg | | | | | | |
| n | 12 | 12 | 12 | 120 | 120 | 120 |
| Mean (SD) | — | — | — | 58.6 (42.0) | 6.96 (3.01) | 3.56 (0.781) |
| GM | 53.2 | 7.00 | 3.43 | 48.6 | 6.44 | 3.49 |
| CV% | 41 | 44 | 22 | 71.6 | 43.3 | 21.9 |

Observed data are from the brentuximab vedotin regulatory submission [30].

*AUC_inf_*, area under the time-concentration curve from time to infinity; *C_max_*, maximum concentration; *CV*, coefficient of variation; *GM*, geometric mean; *MMAE*, monomethyl auristatin E; *SD*, standard deviation; *t_1/2_*, terminal half-life.

^a^Apparent metabolite half-life

**Table S4** Details of study population demographics in the simulated model vs

observed in the clinical trial for enfortumab vedotin

| Parameter | Observed | Simulated |
| --- | --- | --- |
| n | 201 | 2000 |
| Age, y | 65.1 (10.1) | 64.9 (12.2) |
| Weight, kg | 80.3 (20.1) | 72.0 (14.1) |
| Plasma albumin, g/dL | 37.5 (4.1) | 38.2 (7.1) |
| Baseline hematocrit, % | 35.3 (4.76) | 37.6 (4.65) |

Observed data are from the phase 1 trial. Data are mean (standard deviation)

**Table S5** Results of monomethyl auristatin E elimination sensitivity analyses after administration of enfortumab vedotin 1.25 mg/kg

| F_CL_, _Bile_, % | Ketoconazole | | Rifampin | |
| --- | --- | --- | --- | --- |
|  | AUC_last_ ratio | C_max_ ratio | AUC_last_ ratio | C_max_ ratio |
| 60 | 1.48 (1.44–1.52) | 1.18 (1.17–1.19) | 0.40 (0.387–0.419) | 0.66 (0.65–0.68) |
| 70 | 1.38 (1.35–1.41) | 1.15 (1.14–1.16) | 0.47 (0.46–0.49) | 0.72 (0.71–0.73) |
| 80 | 1.29 (1.27–1.31) | 1.12 (1.11–1.13) | 0.59 (0.58–0.59) | 0.80 (0.79–0.80) |

Data expressed as geometric mean ratio (90% confidence interval)

*AUC_last_*, area under the concentration-time curve from time 0 to last quantifiable concentration; *C_max_*, maximum concentration; *F_CL, Bile_*, fraction clearance through the bile

**Fig. S1** Simulated vs observed semilog plasma concentration curves of (**a**) conjugated brentuximab vedotin and (**b**) MMAE following intravenous administration of brentuximab vedotin 1.8 and 2.7 mg/kg. *MMAE*, monomethyl auristatin E

**a**


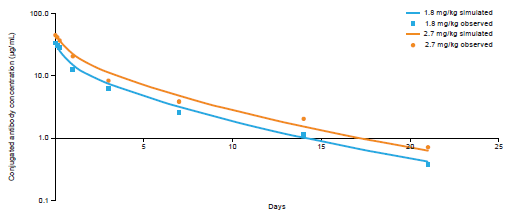


**b**


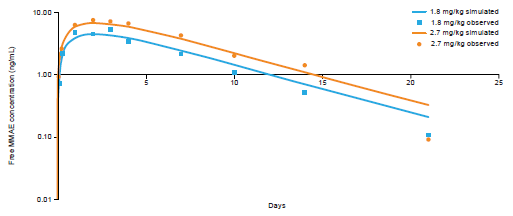


# REFERENCES

- - 1. 1. Adcetris (brentuximab vedotin) Committee for Medicinal Products for Human Use (CHMP) assessment report. Procedure No. EMEA/H/C/002455. 19 July 2012.
    2. 2. Chen Y, Samineni D, Mukadam S, Wong H, Shen B-Q, Lu D, Girish S, Hop C, Yin JY, Li C (2015) Physiologically based pharmacokinetic modeling as a tool to predict drug interactions for antibody-drug conjugates. Clin Pharmacokinet 54 (1):81-93. doi:10.1007/s40262-014-0182-x
    3. 3. Brachet G, Respaud R, Arnoult C, Henriquet C, Dhommee C, Viaud-Massuard MC, Heuze-Vourc'h N, Joubert N, Pugniere M, Gouilleux-Gruart V (2016) Increment in Drug Loading on an Antibody-Drug Conjugate Increases Its Binding to the Human Neonatal Fc Receptor in Vitro. Mol Pharm 13 (4):1405-1412. doi:10.1021/acs.molpharmaceut.6b00082
    4. 4. Sanderson RJ, Hering MA, James SF, Sun MM, Doronina SO, Siadak AW, Senter PD, Wahl AF (2005) In vivo drug-linker stability of an anti-CD30 dipeptide-linked auristatin immunoconjugate. Clin Cancer Res 11 (2 Pt 1):843-852
